# Are prognostic tools losing accuracy? Development and performance of a novel age-calibrated severity scoring system for critically ill patients

**DOI:** 10.1371/journal.pone.0240793

**Published:** 2020-11-04

**Authors:** Rodrigo C. Menezes, Isabella B. B. Ferreira, Thomas A. Carmo, Gabriel P. Telles, Paula L. D. Pugas, Matheus L. Otero, Maria B. Arriaga, Kiyoshi F. Fukutani, Licurgo P. Neto, Sydney Agareno, Nivaldo M. Filgueiras Filho, Kevan M. Akrami, Bruno B. Andrade

**Affiliations:** 1 Hospital de Cidade, Núcleo de Pesquisa, Ensino e Comunicação, Salvador, Brazil; 2 Multinational Organization Network Sponsoring Translational and Epidemiological Research (MONSTER) Initiative, Salvador, Brazil; 3 Instituto Gonçalo Moniz, FIOCRUZ, Salvador, Bahia, Brazil; 4 Faculdade de Medicina da Bahia, Universidade Federal da Bahia, Salvador, Brazil; 5 Universidade do Estado da Bahia (UNEB), Salvador, Bahia, Brazil; 6 Universidade Salvador (UNIFACS), Salvador, Bahia, Brazil; 7 Escola Bahiana de Medicina e Saúde Pública (EBMSP), Salvador, Bahia, Brazil; 8 Hospital de Cidade, Intensive Care Unit, Salvador, Bahia, Brazil; 9 Division of Infectious Diseases and Pulmonary Critical Care and Sleep Medicine, Department of Medicine, University of California, San Diego, California, United States of America; University of Palermo, ITALY

## Abstract

**Objective:**

This study aimed to assess the performance of a commonly used ICU severity score (SAPS3) and determine whether an alternative scoring system may be more accurate across all age strata.

**Methods:**

Retrospective cohort study in a general ICU in Brazil. A secondary analysis was performed with clinical and epidemiological data, present in the first 24 hours of unit admission. Then, a binary logistic regression, followed by cross-validation, was made to develop a novel prognostic tool. ICU mortality was the primary outcome evaluated.

**Results:**

A total of 3042 patients were included over the study period between August 2015 and July 2018 with a median age of 67 ± 18.4 years. SAPS3 performed fairly in prediction of ICU mortality, particularly in the 80 years or older subset. Multivariable regression identified variables independently associated with mortality that were used to develop the Age Calibrated ICU Score (ACIS) tool that performed similarly to SAPS3 across age categories, being slightly superior in the very elderly population (AUC 0.80 vs 0.72).

**Conclusions:**

The ACIS offers a robust and simple tool to predict ICU mortality, particularly in an increasingly elderly critical care population.

## Introduction

The world population is aging with the rate elderly individuals comprising the fastest growing group. By 2050, 16% of the world population is expected to be 65 years or older; moreover, in Latin America, this stratum is expected to double from 56 million to 144 million individuals. As a result of this higher life expectancy and concurrent increased prevalence of comorbidities, patients over 80 years will invariably constitute a greater proportion of intensive care units (ICU) [1]. These patients form a particularly heterogeneous population, with more comorbidities and higher mortality compared to younger patients. Increasingly, modern ICU care teams aims to identify elderly patients who will benefit from treatment in the ICU. In this context, accurate prognosis is paramount to triage resources to those most likely to survive their illness [2–5]. This challenge has become more apparent in the current coronavirus (COVID-19) pandemic, where ICU capacity has become scarce with a surge of critically ill elderly patients. This has left physicians with few tools to accurately determine treatment decisions, often using age as one of the primary factors for ICU admission [6].

The benefit of high cost invasive treatment in elderly patients is unclear. Moreover, prior severity scoring systems have been derived and validated in populations disparate from patients currently admitted to the ICU. Scoring systems, such as the Simplified Acute Physiology Score, have been validated and long used in ICUs around the world to assess the severity of illness. However, these scores did not include an aging population at time of validation, suggesting that their discriminatory capacity may diminish in the coming years with a complex very elderly population [7–9]. This study aims to determine the accuracy of the Simplified Acute Physiology Score (SAPS3) in patients admitted to a general ICU and identify variables associated with mortality to develop an alternative age calibrated scoring system.

## Methods

### Ethics statement

All clinical investigations were conducted according to the principles expressed in the Declaration of Helsinki. The Ethics approval and waiver of consent to participate was approved by the Research Ethics Committee of Hospital Ana Nery under the number 2.571.265 and CAAE 52892315.1.0000.0045.

### Study design and procedures

Observational analytical retrospective cohort study carried conducted from August 2015 to October 2019 in a general ICU of 22 beds in Salvador, Bahia, Brazil. All patients admitted to the ICU with complete data and over 18 years were included. Data was obtained for patient registries and recorded in the Epimed Monitor system.

Covariables included: age, weight, height, sex, comorbidities (arterial hypertension, diabetes, previous myocardial infarction, malignancy, asthma, peripheral vascular disease, structural cardiovascular disease, chronic atrial fibrillation, liver disease, stroke, dementia, alcohol consumption, tobacco consumption, psychiatric disease peptic disease, hypothyroidism, hyperthyroidism, dyslipidemias, reduced level of consciousness, neurological seizures, dependence (independent or minor dependence/bedridden), admission diagnosis, length of ICU and hospital stay, physiologic and laboratory data (lowest mean arterial pressure, highest heart rate, highest respiratory rate, highest temperature, highest leukocyte count, lowest platelets count, highest creatinine, highest arterial lactate, urea and BUN), within the first day of admission, complications, use of vasopressors and mechanical ventilation, ICU mortality and, SAPS3, Charlson Comorbidity Index and MFI scores. The MFI is an instrument to assess the severity of frailty syndrome and is comprised by 11 items [10]. The primary outcome was mortality at ICU discharge.

### Statistical analysis

Categorical variables were expressed as frequencies and percentages and analyzed by Fisher’s exact test or Chi-Squared. Continuous variables with normal distribution were expressed as means (standard deviation, SD) and means between groups were compared with independent T-test. Non-normal continuous variables were expressed as median (interquartile range, IQR) and compared with Mann-Whitney *U* test. Normality was assessed by the D'agostino test. The area under the receiver operating characteristic (AUC) curve was used to determine the discriminate capacity. Probability of ICU death by age was calculated using Kaplan-Meyer curves.

A binary logistic regression, backward stepwise method, was used to identify characteristics independently associated with ICU mortality. Through an analysis of variances, we evaluated the interactions between the study variables and only the interactions with a p <0.05 were entered into regression. The K (10) Fold Cross Validation was performed using Classification And Regression Training package (CARET) available in R [11]. The resampling was performed to evaluate the models on the data sample, using a parameter called “k” that refers to the number of groups the data sample was split into. One proportion of the data was used to discovery the classification and the rest to validate and measure the prediction power of a limited data. Continuous variables were then dichotomized, setting a cut-off value based on the Youden Index J on AUROC analysis. A new regression was made with the dichotomized variables, to identify the adjusted odds ratios and to develop the prognostic tool.

## Results

### Patient characteristics

**Fig 1:**
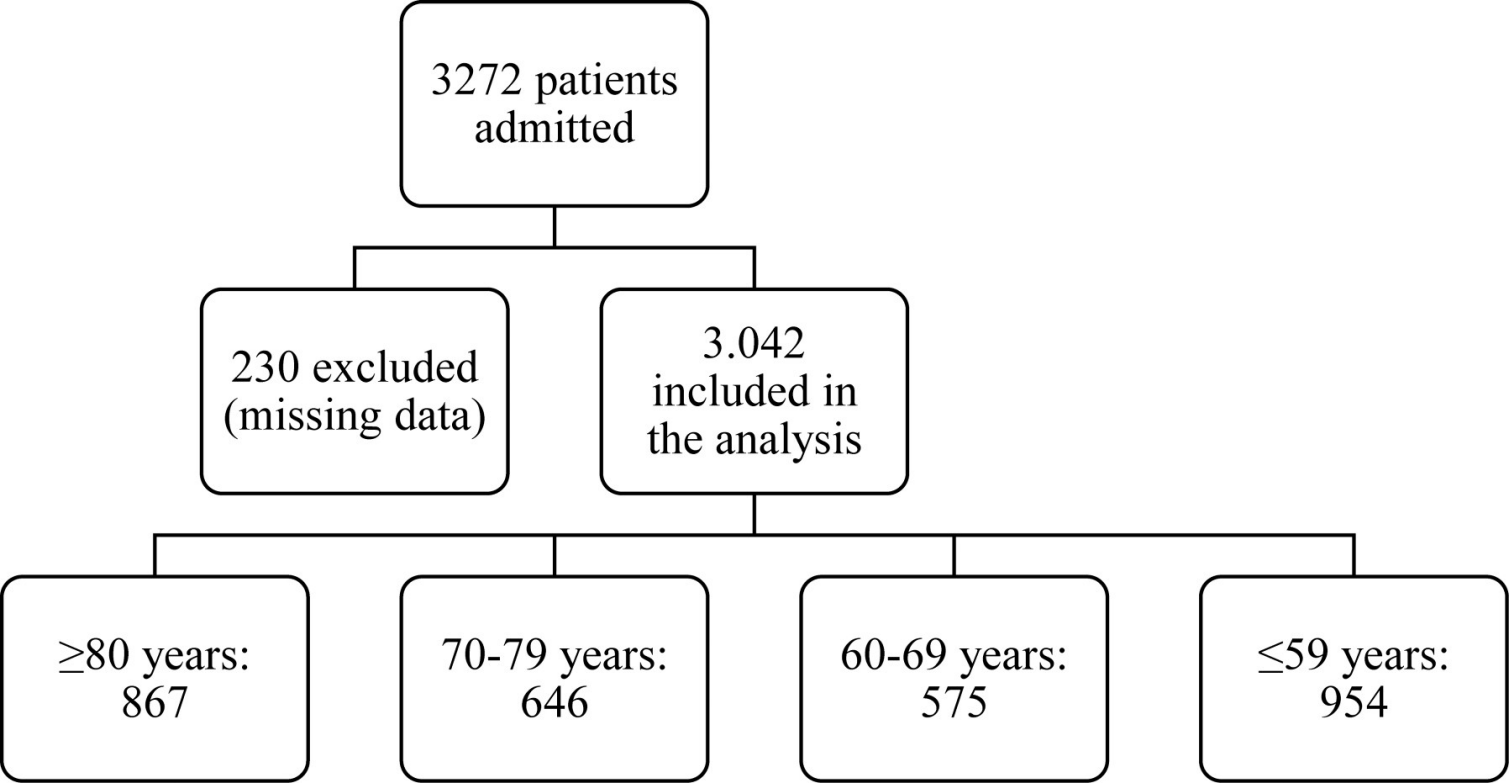
Flowchart of the 3042 patients included in the final analysis.

During the study period, 3,042 patients were admitted to the ICU, with 867 patients 80 years or older, 646 between 70–79, 575 between 60–69 and 954 under 60 (Fig 1). The mean age was 67 ± 18.4 years with a female predominance (53.3%). Eighty-one percent of admissions were non-surgical primarily with cardiovascular, infection/sepsis and neurological diagnoses (21.1%, 17.5% and 16.4% respectively). A total of 463 (15.2%) deaths occurred. The mean value of SAPS3 was 46.2 ± 12.3, corresponding to a predicted mortality of 16.9% with SAPS3. The average ICU length of stay was 7. ± 12.15 days and the average length of hospital stay prior to ICU admission was 2.2 ± 10.2 days (Table 1).

**Table 1:** Characteristics of the study population.

Population Characteristics	General (n = 3042)	≥ 80 yrs. (n = 867)	70–79 yrs. (n = 646)	60–69 yrs. (n = 575)	< 60 yrs. (n = 954)	p-value
Age (years; mean, SD)	67 ± 18.3	87.2 ± 5.1	74.72 ± 2.8	64.67 ± 2.9	44.82 ± 11.6	0.0001
Gender. female (n. %)	1625 (53.4%)	563 (64.9%)	346 (53.6%)	261 (45.4%)	455 (47.7%)	0.0001*
BMI (m/cm²; mean, SD)	25.5 ± 6.1	23.7 ± 5.3	25.8 ± 5.9	26.7 ± 7.3	26.4 ± 5.7	0.0001
ICU Length of Stay (days; mean, SD)	7.6 ± 12.1	8.8 ± 12.2	8.7 ± 14.9	6.9 ± 10.0	6.1 ± 10.8	0.0001
ICU Mortality (n, %)	463 (15.2%)	242 (27.9%)	94 (14.6%)	62 (10.8%)	65 (6.8%)	0.0001*
Hospital Mortality (n, %)	556 (18.2%)	296 (34.1%)	111 (17.1%)	70 (12.1)	79 (8.28%)	0.0001*
**Scores (mean, SD)**						
SAPS3	46.2 ± 12.3	55.5 ± 9	49.8 ± 10.3	43.2 ± 9.7	37.2 ± 10.2	0.0001
Charlson Comorbidity Index	1.5 ± 1.7	1.7 ± 1.6	1.9 ± 1.8	1.8 ± 1.8	1 ± 1.5	0.0001
MFI Score	0.1 ± 0.1	0.19 ± 0.1	0.19 ± 0.11	0.16 ± 0.1	0.09 ± 0.09	0.0001
**Admission Diagnosis (n. %)**						
Cardiovascular	643 (21.1%)	180 (20.8%)	128 (19.8%)	123 (21.4%)	212 (22.2%)	0.704*
Infectious	533 (17.5%)	238 (27.5%)	109 (16.9%)	71 (12.3%)	115 (12.1%)	0.0001*
Surgery	458 (15%)	37 (4.3%)	113 (17.5%)	120 (20.9%)	188 (19.7%)	0.0001*
Neurological or Psychiatric	501 (16.4%)	141 (16.3%)	106 (16.4%)	106 (18.4%)	148 (15.5%)	0.518*
Emergency Surgery	117 (3.8%)	17 (2%)	24 (3.7%)	23 (4%)	53 (5.6%)	0.0018
Others	789 (25.9%)	253 (29.2%)	166 (25.7%)	132 (23%)	238 (24.9%)	0.046*
**Comorbidities (n. %)**						
Dependence	427 (14.1%)	234 (27%)	107 (16.6%)	52 (9.1%)	34 (3.6%)	0.0001*
NYHA 2–4²	197 (7.11%)	75 (8.9%)	58 (9.3%)	31 (5.6%)	33 (4.4%)	0.0001*
Cirrhosis CHILD C³	35 (1.2%)	3 (0.4%)	9 (1.4%)	11 (2%)	12 (1.6%)	0.035*
Malignancy	404 (14.5%)	106 (12.6%)	106 (16.9%)	103 (18.5%)	89 (11.9%)	0.001*
Immunosuppression	48 (1.7%)	9 (1.1%)	8 (1.3%)	8 (1.4%)	23 (3.1%)	0.011*
Cardiac Arrhythmia	298 (10.7%)	133 (15.9%)	93 (14.8%)	32 (5.7%)	40 (5.4%)	0.0001*
Diabetes	1138 (41.1%)	327 (39%)	310 (49.4%)	268 (48%)	233 (31.3%)	0.0001*
Arterial Hypertension	2127 (76.8%)	678 (80.8%)	525 (83.7%)	466 (83.5%)	458 (61.5%)	0.0001*
Stroke	514 (18.5%)	201 (24%)	155 (24.7%)	89 (15.9%)	69 (9.2%)	0.0001*
Dementia	167 (6%)	122 (14.5%)	32 (5.1%)	8 (1.4%)	5 (0.7%)	0.0001*
Tobacco Consumption	207 (7.4%)	46 (5.5%)	44 (7%)	64 (11.5%)	53 (7.1%)	0.0001*
Alcoholism	142 (5.1%)	9 (1.1%)	36 (5.7%)	43 (7.7%)	54 (7.2%)	0.0001*
**Clinical and Laboratory (1st hour, mean, SD)**						
Highest Heart Rate (bpm)	85.9 ± 20.6	85.2 ± 19.8	84.1 ± 20.7	83.2 ± 19.2	89.2 ± 21.5	0.0001
Highest Respiratory Rate (bpm)	20.1 ± 4.4	20.7 ± 4.6	20 ± 4.2	19.5 ± 3.8	19.8 ± 4.5	0.0001
Highest Temperature (°C)	35.8 ± 1.04	35.8 ± 0.9	35.6 ± 1	35.7 ± 1	35.9 ± 1.1	0.0001
Highest Creatinine (mg/dL)	1.42 ± 2.22	1.33 ± 1.65	1.27 ± 1.79	1.43 ± 1.9	1.6 ± 2.9	0.0001
Lowest Platelets Count (uL)	238 ± 111	233 ± 107	241 ± 114	236 ± 104	242 ± 117	0.444
Lowest Mean Arterial Pressure (mmHg)	98 ± 21	96 ± 21	97 ± 21	99 ± 21	98 ± 20	0.042
BUN (mg/dL)	26.4 ± 22.3	31.4 ± 24.1	27.9 ± 23.2	25 ± 18.7	21.8 ± 21.2	0.0001
Highest Arterial Lactate (mmol/L)	2.0 ± 2.3	1.9 ± 2.1	2 ± 2.4	2 ± 2.5	1.9 ± 2.3	0.585
Use of Mechanical Ventilation	108 (12.9%)	86 (13.7%)	71 (12.8%)	126 (13.8%)	108 (12.9%)	0.915*
Use of Vasopressors	247 (8.4%)	74 (8.8%)	57 (9.1%)	52 (9.4%)	64 (7%)	0.322*
Obtunded	790 (26.9%)	332 (38.6%)	192 (29.9%)	124 (21.8%)	142 (15%)	0.0001*

Modified Frailty Index (MFI); Simplified Acute Physiology Score 3 (SAPS3),

*Chi-square test ² New York Heart Association Functional Classification for extent of heart failure. There was a ≈ 9% missing rate for comorbidities.

The 80 years or older subset had a mean age of 87 ± 5.1 years with a predominance of female patients (64.9%). Majority were non-surgical (93.7%) with similar reasons for ICU admission to the general cohort of infection/sepsis, cardiovascular and neurological (27.5%, 20.8% and 16.3% respectively). A total of 247 (27.9%) deaths occurred. The average ICU length of stay was 8.8 ± 12.2 days and the average length hospital stay prior to ICU admission was 2 ± 7.1 days. Additional information can be found in Table 2.

**Table 2:** Characteristics of the population with age of 80 years or older.

Population Characteristics	General (n = 867)	Non-survivors (n = 242)	Survivors (n = 625)	p-value
Age (years; mean, SD)	87.2 ± 5.1	87.7 ± 5.1	87 ± 5.1	0.50
Gender. female (n. %)	563 (64.9%)	144 (59.5%)	419 (67%)	0.037*
BMI (m/cm²; mean, SD)	23.6 ± 5.3	22.2 ± 5.4	24.2 ± 5.1	0.0001
Unit Length of Stay (days; mean, SD)	8.8 ± 12.2	13.8 ± 19.1	6.9 ± 7.34	0.0001
Readmission (n, %)	64 (7.3%)	28 (11.5%)	36 (5.7%)	0.003*
**Scores (mean, SD)**				
Saps3	55.5 ± 9	61.1 ± 10.7	53.4 ± 7.3	0.0001
Charlson Comorbidity Index	0.1 ± 0.1	0.21 ± 0.11	0.18 ± 0.11	0.0001
MFI Score	1.7 ± 1.6	2 ± 1.	1.5 ± 1.5	0.003
**Admission Diagnosis (n. %)**				
Cardiovascular	180 (20.7%)	36 (14.8%)	144 (23%)	0.008*
Infectious	238 (27.4%)	91 (37.6%)	147 (23.5%)	0.0001*
Surgery	37 (4.2%)	3 (1.2%)	34 (5.4%)	0.006*
Neurological or Psychiatric	141 (16.2%)	36 (14.8%)	105 (16.8%)	0.485*
Emergency Surgery	17 (1.9%)	5 (2%)	12 (1.9%)	0.892*
Others	253 (29.2%)	71 (29.3%)	182 (29.1%)	0.960*
**Comorbidities (n. %)**				
Dependence	234 (27%)	104 (42.9%)	130 (20.8%)	0.0001*
Heart Failure	75 (8.9%)	18 (7.7%)	57 (9.3%)	0.473*
Hepatic Failure	3 (0.3%)	2 (0.8%)	1 (0.1%)	0.128*
Renal Failure	86 (10.2%)	29 (12.5%)	57 (9.3%)	0.175*
Malignancy	106 (12.6%)	43 (18.6%)	63 (10.3%)	0.001*
Immunosuppression	9 (1%)	3 (1.3%)	6 (0.9%)	0.695*
Cardiac Arrhythmia	133 (15.8%)	36 (15.5%)	97 (15.9%)	0.896*
Diabetes	327 (38.9%)	95 (41.1%)	232 (38.1%)	0.431*
Arterial Hypertension	678 (80.8%)	177 (76.6%)	501 (82.4%)	0.058*
Stroke	201 (23.9%)	61 (26.4%)	140 (23%)	0.305*
Dementia	122 (14.5%)	49 (21.2%)	73 (12%)	0.001*
Tobacco Consumption	46 (5.4%)	10 (4.3%)	36 (5.9%)	0.366*
Alcoholism	9 (1%)	3 (1.3%)	6 (0.9%)	0.695*
**Clinical and Laboratory (1st hour, mean, SD)**				
Highest Heart Rate (bpm)	85.2 ± 19.8	91.7 ± 20.5	82.8 ± 19	0.0001
Highest Respiratory Rate (bpm)	20.7 ± 4.6	21.6 ± 4.7	20.4 ± 4.5	0.0001
Highest Temperature (°C)	35.8 ± 0.9	35.7 ± 1	35.8 ± 0.8	0.185
Highest Creatinine (mg/dL)	1.3 ± 1.65	1.62 ± 1.9	1.21 ± 1.5	0.026
Lowest Platelets Count (μL)	233 ± 107	236 ± 117	232 ± 103.2	0.892
Lowest mean arterial pressure (mmHg)	96.9 ± 21.4	94.4 ± 22.3	97.8 ± 21	0.026
BUN (mg/dL)	31.4 ± 24.1	40.2 ± 31.5	28. ± 19.5	0.0001
Highest Arterial Lactate (mmol/L)	1.9 ± 2.1	2.8 ± 3.2	1.6 ± 1.1	0.0001
Use of mechanical ventilation	108 (12.9%)	69 (28.9%)	39 (6.5%)	0.0001*
Use of Vasopressors	74 (8.8%)	50 (21%)	24 (4%)	0.0001*
Obtunded	332 (38.6%)	140 (58.5%)	192 (30.9%)	0.0001*

Modified Frailty Index (MFI); Simplified Acute Physiology Score 3 (SAPS3),

*Chi-square test. There was a ≈ 9% missing rate for comorbidities. Yrs: years.

### Comparison between non-survivors and survivors

In the whole population, those who died had a mean age of 76.2 ± 15.6 years with a female predominance (52.4%) compared with survivors whose mean age of 65.3 ± 18.3 years and a predominance of females (53.5%). The non-survivors presented with lower BMI, longer ICU length of stay, higher admission SAPS3, Charlson Comorbidity Index (CCI) and Modified Frailty Index (MFI) scores. These and other physiological data can be found in the S1 Table.

The non-survivors in the 80 years or older subgroup did not vary in their mean age compared to survivors (87.7 ± 5.1 vs 87 ± 5.1 year, respectively). Non-survivors had lower Body Mass Index (BMI), ICU length of stay, and higher SAPS3, CCI and MFI scores values, as seen in Table 2. Data regarding other age strata are summarized in the S2 Table.

### Derivation of a novel severity score

**Fig 2:**
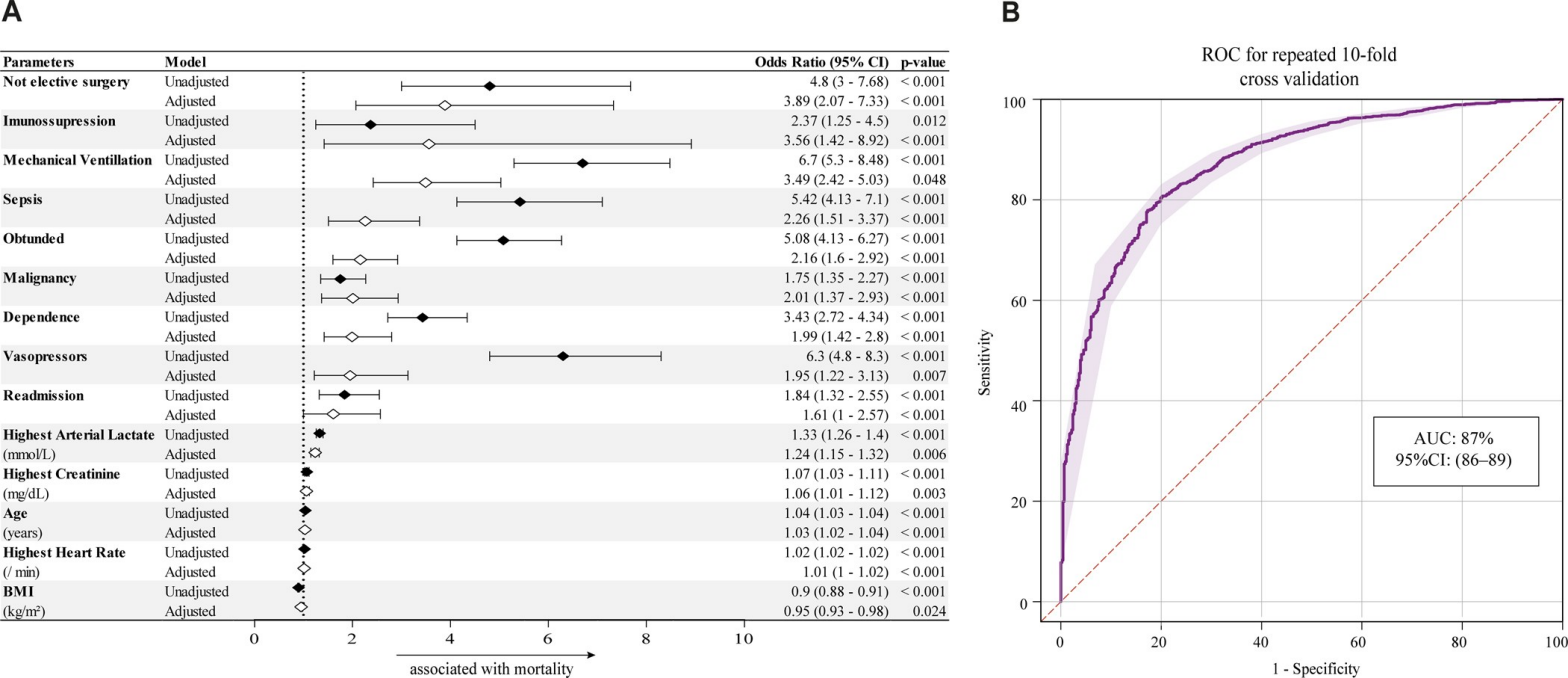
(A) Adjusted and unadjusted binary regression model for ICU mortality. Univariable analysis yielded unadjusted odds of death. Multivariable regression adjusted for differences in baseline characteristics (variables of p<0.1 identified in univariable analysis). (B) ROC curve after K-10 fold-validation, showing model accuracy.

**Fig 3:**
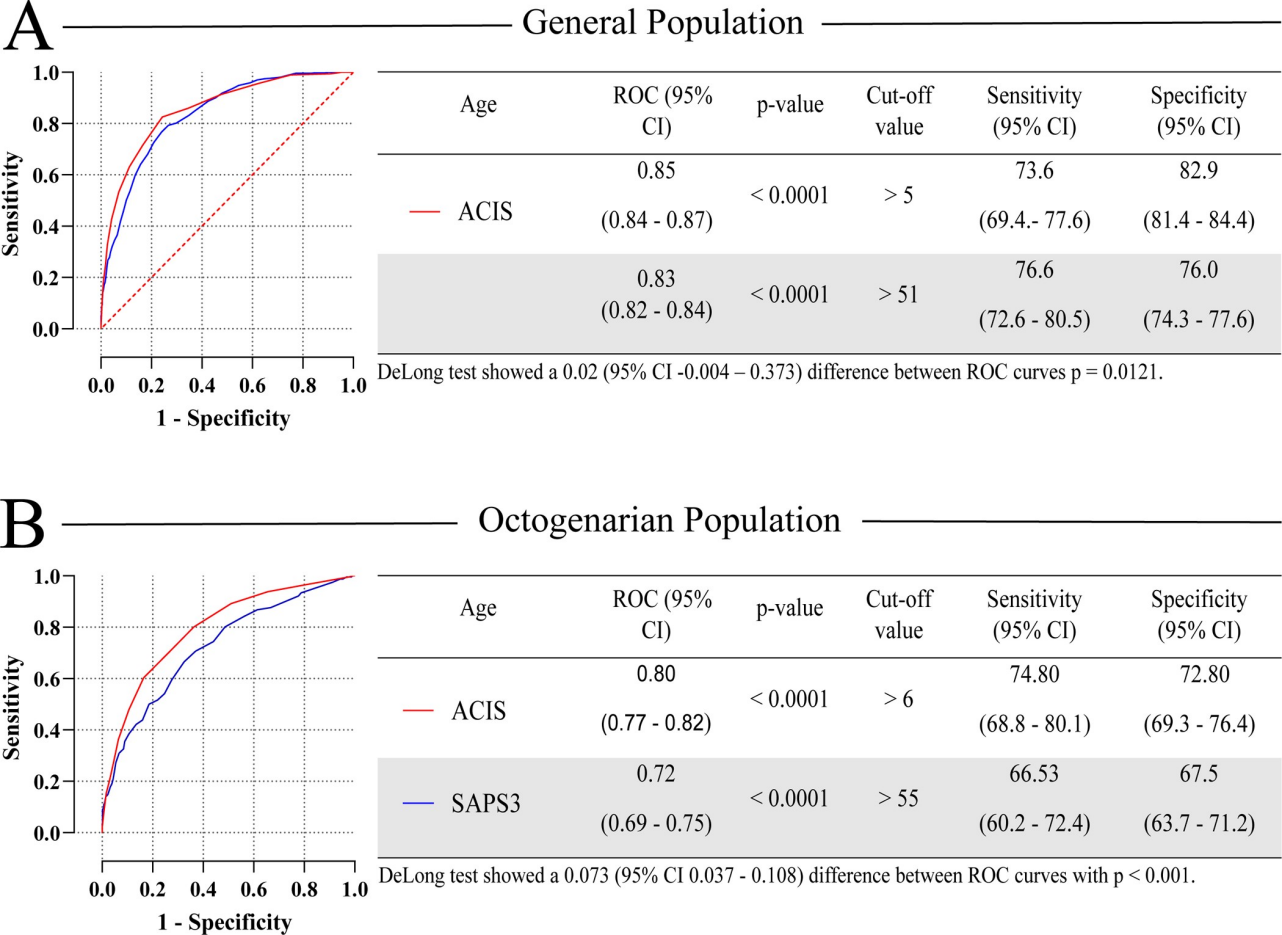
Comparison between SAPS3 and ACIS receiver operating characteristic (ROC) curves for prediction of ICU mortality on ICU general population (A) and 80 years or older population (B). The ACIS novel score outperformed the SAPS3 in the 80 years or older subset with similar discriminate function in the other age strata.

Multivariable regression analysis yielded 14 variables that were used to develop an Age Calibrated ICU Score (ACIS) and a punctuation was assigned to each variable based on their adjusted OR values (Fig 2). Then, an approximation to an integer was made to facilitate usability, without significant compromise of its AUC. The ACIS is a score where the following variables are present or absent during admission and they add up to a maximum of 27 points: patient with minor dependence or bedridden (2 points); sepsis (2 points); need of vasopressor (3 points), need of mechanical ventilation (3 points) Glasgow lower than 15 (2 points), immunosuppression (2 points), malignancy (2 points), arterial lactate above 2.64 mmol/L (2 points), serum creatinine above 1.2 (2 points), being 80 years or older (2 points), heart rate over 100 beats per minute (2 points), BMI less than 23 (1 points), being readmitted 24 hours after ICU discharge (2 points), being admitted after elective surgery (-3 points). There was a small, difference in discriminate function between SAPS3 and ACIS in the general ICU population (AUC 0.83 CI 95% 0.82 to 0.84) vs AUC 0.85 (95% CI 0.84 to 0.87) (Fig 3A). When stratified by age, SAPS3 performed fairly in prediction of ICU mortality in the 80 years or older population with an AUC of 0.72 (CI 95% 0.69 to 0.75; P < 0.0001). In contrast, the ACIS discriminate function was significantly, but slightly, superior to SAPS3 in the 80 years or older population (AUC 0.80 vs 0.72, P < 0.001) as demonstrated in Fig 3B. The accuracy of SAPS3 and ACIS in the other age strata is shown S1 Fig. ACIS presented a performance slightly superior to SAPS3 in the age group 80 years or older, this difference being statistically significant, and a similar performance in the other age groups (S3 Table). When comparing the predictive capacity of both scores for hospital mortality, SAPS3 and ACIS had similar performance in the general population (AUC 0.84 vs. 0.84 p = 0.75) and a difference in the 80 years or older group (AUC 0.73 vs. 0.78; p = 0.004) (S4 Table) and 59 years or younger group (0.90 vs. 0.86; p = 0.05), the last not being statistically significant.

## Discussion

This study evaluated the accuracy of the SAPS3 severity score in a large heterogeneous ICU cohort comprised of very elderly patients. In the general population, SAPS3 demonstrated good accuracy in prediction of ICU mortality and it performed fairly in the very elderly subset of our critically ill cohort. Prior studies have demonstrated similar reductions in the accuracy of SAPS3 and other severity scores in elderly patients admitted to the ICU [9]. Notably, the novel ACIS tool outperformed the SAPS3 in our 80 years or older population and most other age strata.

The ACIS novel scoring system is a practical bedside tool that determines disease severity and subsequent ICU mortality in the general and 80 years or older ICU population. Furthermore, the ACIS offers an alternative severity scoring system in clinical trials to determine study inclusion and to compare the severity of enrolled individuals with future or prior randomized studies [12–14]. In the absence of an accurate tool to determine the severity of those admitted to the ICU, critical care trials will continue to be burdened by misclassified heterogeneous populations. Inaccuracy of commonly used ICU scores is an underappreciated reality in both clinical management of those admitted to the ICU and interpretation of results from randomized clinical trials, particularly in the 80 years or older subset.

Factors associated with survival, rather than mortality in our cohort also revealed that patients from 60–69 years of age who were independent prior to admission with a BMI classification as overweight (25–29) had a higher probability of survival.

While our large cohort study confirmed the reasonable performance of SAPS3 in elderly ICU patients and derived the ACIS tool, there are several limitations that must be acknowledged. First, as a single center study there may be unknown factors related to the ICU population impacting the poor performance of SAPS3 or improved discriminate function of the ACIS novel system in our elderly subset. Given the retrospective collection of all relevant clinical data, there was a limitation due to unavailability or inaccessibility to variables that were not available in our electronic record, such as some laboratory tests and palliative care. Due to our relatively large cohort of 80 years or older, patient characteristics are unlikely to affect the performance of SAPS3 or ACIS. Second, as a study conducted in a tertiary ICU in an urban setting in Brazil, there may be local epidemiologic factors that could interfere with generalizability in the wider ICU population. While severity scores such as SAPS3 have been extensively studied in Europe and in the United States, this study represents one of the first performed in a resource limited setting to specifically evaluate the performance of SAPS3 and derive a novel severity tool. Our findings call into question the routine use of SAPS3 in the ICU, particularly among elderly patients whose prognosis may be inaccurately determined. Lastly, emerging evidence suggests that it may be the change in severity scores over time from admission that more accurately predicts ICU mortality.

In conclusion, the ACIS described here offers a robust and simple alternative to existing severity scores to predict ICU mortality and aid in triage, particularly in an increasingly elderly critical care population.

## Supporting information

S1 Table General survivors and non-survivors’ characteristics. (DOCX)

S2 Table Study population characteristics. (DOCX)

S3 Table Comparison of ACIS and SAPS3 stratified by age. (DOCX)

S4 Table Comparison of ACIS and SAPS3 stratified by age for hospital mortality. (DOCX)

S1 Fig Comparison between SAPS3 (A) and ACIS (B) receiver operating characteristic (ROC) curves stratified by age was performed to test accuracy of the indicated scores in prediction of ICU mortality. Discriminate function of the ACIS was superior to SAPS3 in the 80 years or older subset with comparable performance in the other age interval
(TIF)

S2 Fig Modified Kaplan-Meier analysis for an ACIS cutoff of 5 points. Survival probability is significantly decreased in those over 75 years old with an ACIS score ≥5.
(TIF)

S3 Fig Determination of cut-off values using the associated Youden criterion on the ROC curve. (TIF)
