# Supplementary material for: Are prognostic tools losing accuracy? Development and performance of a novel age-calibrated severity scoring system for critically ill patients
Source: PLoS One. 2020 Nov 4;15(11):e0240793. doi: 10.1371/journal.pone.0240793 (PMC7641388; doi:10.1371/journal.pone.0240793)
Supplement: S1 Table — (DOCX) [file pone.0240793.s001.docx]

S1 Table. General survivors and non-survivors’ characteristics

| Population Characteristics | Non-Survivors  (n = 463) | Survivors  (n = 2579) | p-value |
| --- | --- | --- | --- |
| Age (mean ± SD) | 76.24 ± 15.69 | 65.34 ± 18.35 | 0.0001 |
| Gender. female (n. %) | 243 (52.48%) | 1382 (53.59%) | 0.661* |
| BMI (mean ± SD) | 23.01 ± 5.68 | 25.99 ± 6.07 | 0.0001 |
| Length Hospital Stay Prior Unit Admission (mean ± SD) | 3.51 ± 10.04 | 2.01 ± 10.31 | 0.005 |
| Unit Length of Stay (mean ± SD) | 14.87 ± 22.14 | 6.32 ± 8.66 | 0.0001 |
| Readmission (n. %) | 52 (11.23%) | 166 (6.44%) | 0.0001* |
| **Scores (n, SD)** |  |  |  |
| Saps3 | 59.22 ± 11.71 | 43.95 ± 10.89 | 0.0001 |
| Charlson Comorbidity Index | 2.1 ± 2.01 | 1.46 ± 1.67 | 0.0001 |
| MFI Score | 0.18 ± 0.12 | 0.14 ± 0.11 | 0.0001 |
| **Admission Diagnosis (n. %)** |  |  |  |
| Cardiovascular | 77 (16.63%) | 566 (21.96%) | 0.010* |
| Infectious | 156 (33.69%) | 377 (14.63%) | 0.0001* |
| Surgery | 19 (4.1%) | 439 (17.04%) | 0.0001* |
| Neurological or Psychiatric | 71 (15.33%) | 430 (16.69%) | 0.471* |
| Emergency Surgery | 11 (2.38%) | 106 (4.11%) | 0.074* |
| Others | 129 (27.86%) | 660 (25.61%) | 0.309* |
| **Comorbidities (n. %)** |  |  |  |
| Dependence | 140 (30.24%) | 287 (11.2%) | 0.0001* |
| Heart Failure | 27 (6.18%) | 170 (7.29%) | 0.407* |
| Hepatic Failure | 10 (2.29%) | 25 (1.07%) | 0.037* |
| Renal Failure | 57 (13.04%) | 270 (11.58%) | 0.384* |
| Malignancy | 93 (21.28%) | 311 (13.34%) | 0.0001* |
| Immunosuppression | 14 (3.2%) | 34 (1.46%) | 0.010* |
| Cardiac Arrhythmia | 58 (13.27%) | 240 (10.29%) | 0.065* |
| Diabetes | 186 (42.56%) | 952 (40.82%) | 0.498* |
| Arterial Hypertension | 331 (75.74%) | 1796 (77.02%) | 0.563* |
| Cardiovascular Disease | 66 (15.1%) | 405 (17.37%) | 0.248* |
| Stroke | 102 (23.29%) | 412 (17.63%) | 0.005* |
| Dementia | 59 (13.5%) | 108 (4.63%) | 0.0001* |
| Tobacco Consumption | 25 (5.72%) | 182 (7.8%) | 0.129* |
| Alcoholism | 26 (5.95%) | 116 (4.97%) | 0.396* |
| **Clinical and Laboratory (1st hour. mean. SD)** |  |  |  |
| Highest Heart Rate (bpm) | 94.16 ± 22.87 | 84.45 ± 19.83 | 0.0001 |
| Highest Respiratory Rate (bpm) | 21.7 ± 5.2 | 19.82 ± 4.22 | 0.0001 |
| Highest Temperature (°C) | 35.78 ± 1.15 | 35.8 ± 1.02 | 0.308 |
| Highest Creatinine (mg/dL) | 1.79 ± 2.15 | 1.36 ± 2.23 | 0.0001 |
| Lowest Platelets Count (uL) | 231.75 ± 129.83 | 239.84 ± 108.16 | 0.005 |
| Mean Arterial Pressure (mmHg) | 93.91 ± 22.73 | 99.08 ± 20.59 | 0.0001 |
| Highest Arterial Lactate (mmol/L) | 3.46 ± 4.50 | 1.70 ± 1.45 | 0.0001 |
| BUN (mg/dL) | 40.26 ± ± 31.07 | 23.98 ± 19.42 | 0.0001 |
| **Complications (n. %)** |  |  |  |
| Use of mechanical ventilation | 176 (38.94%) | 215 (8.68%) | 0.0001* |
| Use of Vasopressors | 117 (25.88%) | 130 (5.25%) | 0.0001* |
| Obtunded | 260 (57.02%) | 530 (20.68%) | 0.0001* |

Modified Frailty Index (MFI); Simplified Acute Phisiology Score 3 (SAPS3)

*Chi-square test
