# Supplementary material for: Are prognostic tools losing accuracy? Development and performance of a novel age-calibrated severity scoring system for critically ill patients
Source: PLoS One. 2020 Nov 4;15(11):e0240793. doi: 10.1371/journal.pone.0240793 (PMC7641388; doi:10.1371/journal.pone.0240793)
Supplement: S2 Table — (DOCX) [file pone.0240793.s002.docx]

**S2 Table.** Study population characteristics.

| Characteristics | Age range | | | | | | | |
| --- | --- | --- | --- | --- | --- | --- | --- | --- |
|  | 80+ | | 70 - 79 | | 60 - 69 | | < 50 | |
|  | Non-survivor  (n = 242) | Survivor  (n = 625) | Non-survivor  (n = 94) | Survivor  (n = 552) | Non-survivor  (n = 62) | Survivor  (n = 513) | Non-survivor  (n = 65) | Survivor  (n = 889) |
| Age (years; mean ± SD) | 87.72 ± 5.16 | 87 ± 5.1 | 74.89 ± 2.83 | 74.7 ± 2.9 | 64.44 ± 2.96 | 64.69 ± 2.92 | 46.66 ± 11.48 | 44.69 ± 11.69 |
| Gender (n, %) | 144 (59.5%) | 419 (67.04%) | 46 (48.94%) | 300 (54.35%) | 31 (50%) | 230 (44.83%) | 22 (33.85%) | 433 (48.71%) |
| BMI (m/cm²; mean ± SD) | 22.24 ± 5.47 | 24.23 ± 5.18 | 23.68 ± 5.56 | 26.09 ± 5.85 | 23.71 ± 4.92 | 27.07 ± 7.46 | 24.29 ± 6.89 | 26.55 ± 5.63 |
| Length Hospital Stay Prior Unit Admission (days; mean ± SD) | 3.32 ± 10.06 | 1.59 ± 5.6 | 3.47 ± 8.32 | 2.04 ± 7.32 | 2.41 ± 7.26 | 2.03 ± 7.64 | 5.31 ± 13.78 | 2.26 ± 14.8 |
| Unit Length Stay (days; mean ± SD) | 13.85 ± 19.13 | 6.9 ± 7.34 | 17.84 ± 29.71 | 7.23 ± 9.78 | 14.76 ± 17.64 | 6.01 ± 8.23 | 14.49 ± 23.74 | 5.52 ± 8.94 |
| Readmission (n, %) | 28 (11.57%) | 36 (5.76%) | 13 (13.83%) | 37 (6.7%) | 5 (8.06%) | 36 (7.02%) | 6 (9.23%) | 57 (6.41%) |
| **Scores (mean ± SD)** |  |  |  |  |  |  |  |  |
| Saps3 | 61.11 ± 10.71 | 53.42 ± 7.33 | 60.46 ± 12.8 | 48.02 ± 8.66 | 55.13 ± 11.55 | 41.8 ± 8.47 | 54.31 ± 11.7 | 36 ± 8.92 |
| Charlson Comorbidity Index | 2.07 ± 1.91 | 1.58 ± 1.57 | 2.39 ± 2.15 | 1.86 ± 1.74 | 2.26 ± 2.12 | 1.75 ± 1.83 | 1.58 ± 2 | 0.96 ± 1.49 |
| MFI Score | 0.21 ± 0.11 | 0.18 ± 0.11 | 0.2 ± 0.12 | 0.18 ± 0.11 | 0.15 ± 0.12 | 0.16 ± 0.1 | 0.09 ± 0.09 | 0.09 ± 0.09 |
| **Admission Diagnosis (n, %)** |  |  |  |  |  |  |  |  |
| Cardiovascular | 36 (14.88%) | 144 (23.08%) | 13 (13.83%) | 115 (20.87%) | 17 (27.42%) | 106 (20.66%) | 11 (16.92%) | 201 (22.61%) |
| Infeccious | 91 (37.6%) | 147 (23.56%) | 32 (34.04%) | 77 (13.97%) | 12 (19.35%) | 59 (11.5%) | 21 (32.31%) | 94 (10.57%) |
| Surgery | 3 (1.24%) | 34 (5.45%) | 11 (11.7%) | 102 (18.51%) | 4 (6.45%) | 116 (22.61%) | 1 (1.54%) | 187 (21.03%) |
| Neurological or Psychiatric | 36 (14.88%) | 105 (16.83%) | 10 (10.64%) | 96 (17.42%) | 10 (16.13%) | 96 (18.71%) | 15 (23.08%) | 133 (14.96%) |
| Emergency Surgery | 5 (2.07%) | 12 (1.92%) | 2 (2.13%) | 22 (3.99%) | 2 (3.23%) | 21 (4.09%) | 2 (3.08%) | 51 (5.74%) |
| Others | 71 (29.34%) | 182 (29.17%) | 26 (27.66%) | 140 (25.41%) | 17 (27.42%) | 115 (22.42%) | 15 (23.08%) | 223 (25.08%) |
| **Comorbidities (n, %)** |  |  |  |  |  |  |  |  |
| Dependence | 104 (42.98%) | 130 (20.83%) | 26 (27.66%) | 81 (14.7%) | 7 (11.29%) | 45 (8.81%) | 3 (4.62%) | 31 (3.53%) |
| Heart Failure | 18 (7.79%) | 57 (9.38%) | 5 (5.62%) | 53 (9.85%) | 2 (3.45%) | 29 (5.8%) | 2 (3.39%) | 31 (4.52%) |
| Hepatic Failure | 2 (0.87%) | 1 (0.16%) | 2 (2.25%) | 7 (1.3%) | 4 (6.9%) | 7 (1.4%) | 2 (3.39%) | 10 (1.46%) |
| Renal Failure | 29 (12.55%) | 57 (9.38%) | 14 (15.73%) | 55 (10.22%) | 7 (12.07%) | 76 (15.2%) | 7 (11.86%) | 82 (11.95%) |
| Malignancy | 43 (18.61%) | 63 (10.36%) | 22 (24.72%) | 84 (15.61%) | 15 (25.86%) | 88 (17.6%) | 13 (22.03%) | 76 (11.08%) |
| Immunossupression | 3 (1.3%) | 6 (0.99%) | 4 (4.49%) | 4 (0.74%) | 2 (3.45%) | 6 (1.2%) | 5 (8.47%) | 18 (2.62%) |
| Cardiac Arrhythmia | 36 (15.58%) | 97 (15.95%) | 12 (13.48%) | 81 (15.06%) | 6 (10.34%) | 26 (5.2%) | 4 (6.78%) | 36 (5.25%) |
| Diabetes | 95 (41.13%) | 232 (38.16%) | 47 (52.81%) | 263 (48.88%) | 27 (46.55%) | 241 (48.2%) | 17 (28.81%) | 216 (31.49%) |
| Arterial Hypertension | 177 (76.62%) | 501 (82.4%) | 72 (80.9%) | 453 (84.2%) | 46 (79.31%) | 420 (84%) | 36 (61.02%) | 422 (61.52%) |
| Cardiovascular Disease | 37 (16.02%) | 123 (20.23%) | 11 (12.36%) | 93 (17.29%) | 10 (17.24%) | 87 (17.4%) | 8 (13.56%) | 102 (14.87%) |
| Stroke | 61 (26.41%) | 140 (23.03%) | 25 (28.09%) | 130 (24.16%) | 13 (22.41%) | 76 (15.2%) | 3 (5%) | 66 (9.55%) |
| Dementia | 49 (21.21%) | 73 (12.01%) | 6 (6.74%) | 26 (4.83%) | 1 (1.72%) | 7 (1.4%) | 3 (5.08%) | 2 (0.29%) |
| Tobacco Consumption | 10 (4.33%) | 36 (5.92%) | 10 (11.24%) | 34 (6.32%) | 2 (3.45%) | 62 (12.4%) | 3 (5.08%) | 50 (7.29%) |
| Alcoholism | 3 (1.3%) | 6 (0.99%) | 10 (11.24%) | 26 (4.83%) | 3 (5.17%) | 40 (8%) | 10 (16.95%) | 44 (6.41%) |
| **Clinical and Laboratory (1^st^ hour; mean, SD)** |  |  |  |  |  |  |  |  |
| Highest Heart Rate (bpm) | 91.75 ± 20.54 | 82.8 ± 19.04 | 95.4 ± 24.5 | 82.27 ± 19.45 | 93.15 ± 24.58 | 82.08 ± 18.1 | 102.34 ± 25.46 | 88.33 ± 20.98 |
| Highest Respiratory Rate (bpm) | 21.63 ± 4.72 | 20.46 ± 4.59 | 21.97 ± 6.04 | 19.76 ± 3.82 | 20.87 ± 4.18 | 19.37 ± 3.77 | 22.36 ± 6.36 | 19.67 ± 4.37 |
| Highest Temperature (°C) | 35.74 ± 1.05 | 35.86 ± 0.87 | 35.58 ± 1.14 | 35.69 ± 1.05 | 35.78 ± 1.13 | 35.71 ± 1.02 | 36.21 ± 1.39 | 35.89 ± 1.1 |
| Highest Creatinine (mg/dL) | 1.62 ± 1.96 | 1.21 ± 1.5 | 2.01 ± 2.42 | 1.14 ± 1.63 | 1.59 ± 1.5 | 1.41 ± 1.97 | 2.26 ± 2.83 | 1.56 ± 2.99 |
| Lowest Platelets Count (uL) | 236.67 ± 117.73 | 232.09 ± 103.28 | 241.53 ± 149.99 | 241.77 ± 107.97 | 229.98 ± 124.03 | 237.7 ± 101.79 | 201.73 ± 144.24 | 245.51 ± 115.01 |
| Mean Arterial Pressure (mmHg) | 94.4 ± 22.38 | 97.88 ± 21.01 | 91.25 ± 24.64 | 99.04 ± 20.68 | 93.12 ± 20.4 | 100.68 ± 21.22 | 96.73 ± 23.39 | 99.03 ± 19.83 |
| BUN (mg/dL) | 40.27 ± 31.52 | 28.07 ± 19.58 | 44.62 ± 34.02 | 25.01 ± 19.42 | 32.98 ± 19.33 | 24.07 ± 18.4 | 41.05 ± 33.61 | 20.43 ± 19.3 |
| Highest Arterial Lactate (mmol/L) | 2.8 ± 3.27 | 1.6 ± 1.19 | 3.87 ± 4.94 | 1.71 ± 1.47 | 4.53 ± 5.88 | 1.74 ± 1.41 | 4.32 ± 5.78 | 1.77 ± 1.62 |
| **Complications (n, %)** |  |  |  |  |  |  |  |  |
| Mechanical Ventilation | 69 (28.99%) | 39 (6.51%) | 41 (46.07%) | 45 (8.33%) | 31 (50.82%) | 40 (8.13%) | 35 (54.69%) | 91 (10.76%) |
| Vasopressors | 50 (21.01%) | 24 (4.01%) | 26 (29.21%) | 31 (5.74%) | 20 (32.79%) | 32 (6.5%) | 21 (32.81%) | 43 (5.08%) |
| Obtunded | 140 (58.58%) | 192 (30.92%) | 52 (56.52%) | 140 (25.45%) | 36 (59.02%) | 88 (17.29%) | 32 (50%) | 110 (12.46%) |

Modified Frailty Index (MFI); Simplified Acute Phisiology Score 3 (SAPS3)
