# Supplementary material for: Are prognostic tools losing accuracy? Development and performance of a novel age-calibrated severity scoring system for critically ill patients
Source: PLoS One. 2020 Nov 4;15(11):e0240793. doi: 10.1371/journal.pone.0240793 (PMC7641388; doi:10.1371/journal.pone.0240793)
Supplement: S3 Table — (DOCX) [file pone.0240793.s003.docx]

**S3 Table.** Comparison of ACIS and SAPS3 stratified by age.

| Age | AUROC (95% CI) | Difference between areas* | p-value |
| --- | --- | --- | --- |
| **80 years or older** |  |  |  |
| ACIS | 0.80 (0.77 - 0.82) | 0.07 (0.038 - 0.108) | **< 0.001** |
| SAPS3 | 0.72 (0.69 - 0.75) |  |  |
| **70 to 79** |  |  |  |
| ACIS | 0.84 (0.80 - 0.86) | 0.04 (0.0004 - 0.081) | 0.05 |
| SAPS3 | 0.79 (0.76 - 0.82) |  |  |
| **60 to 69** |  |  |  |
| ACIS | 0.84 (0.81 - 0.87) | 0.03 (-0.033 - 0.053) | 0.64 |
| SAPS3 | 0.83 (0.80 - 0.86) |  |  |
| **59 years or younger** |  |  |  |
| ACIS | 0.87 (0.85 - 0.90) | 0.03 (-0.015 - 0.078) | 0.18 |
| SAPS3 | 0.91 (0.89 - 0.92) |  |  |

*DeLong et al, 1988
