# Supplementary material for: Are prognostic tools losing accuracy? Development and performance of a novel age-calibrated severity scoring system for critically ill patients
Source: PLoS One. 2020 Nov 4;15(11):e0240793. doi: 10.1371/journal.pone.0240793 (PMC7641388; doi:10.1371/journal.pone.0240793)
Supplement: S4 Table — (DOCX) [file pone.0240793.s004.docx]

**S4 Table.** Comparison of ACIS and SAPS3 stratified by age for hospital mortality.

| Age | AUROC (95% CI) | Difference between areas* | p-value |
| --- | --- | --- | --- |
| **General** |  |  |  |
| ACIS | 0.84 (0.82 - 0.85) | 0.002 (-0.014 - 0.019) | 0.31 |
| SAPS3 | 0.84 (0.82 - 0.85) |  |  |
| **80 years or older** |  |  |  |
| ACIS | 0.78 (0.74 - 0.81) | 0.05 (0.016 - 0.085) | **0.004** |
| SAPS3 | 0.73 (0.70 - 0.76) |  |  |
| **70 to 79** |  |  |  |
| ACIS | 0.79 (0.76 - 0.83) | 0.01 (-0.025 - 0.056) | 0.44 |
| SAPS3 | 0.78 (0.75 - 0.81) |  |  |
| **60 to 69** |  |  |  |
| ACIS | 0.83 (0.79 - 0.86) | 0.004 (-0.041 - 0.049) | 0.17 |
| SAPS3 | 0.83 (0.79 - 0.86) |  |  |
| **59 years or younger** |  |  |  |
| ACIS | 0.86 (0.83 - 0.89) | 0.03 (-0.0006 - 0.090) | 0.05 |
| SAPS3 | 0.90 (0.87 - 0.91) |  |  |

*DeLong et al, 1988
