# Supplementary figures and images for: Are prognostic tools losing accuracy? Development and performance of a novel age-calibrated severity scoring system for critically ill patients
Source: PLoS One. 2020 Nov 4;15(11):e0240793. doi: 10.1371/journal.pone.0240793 (PMC7641388; doi:10.1371/journal.pone.0240793)

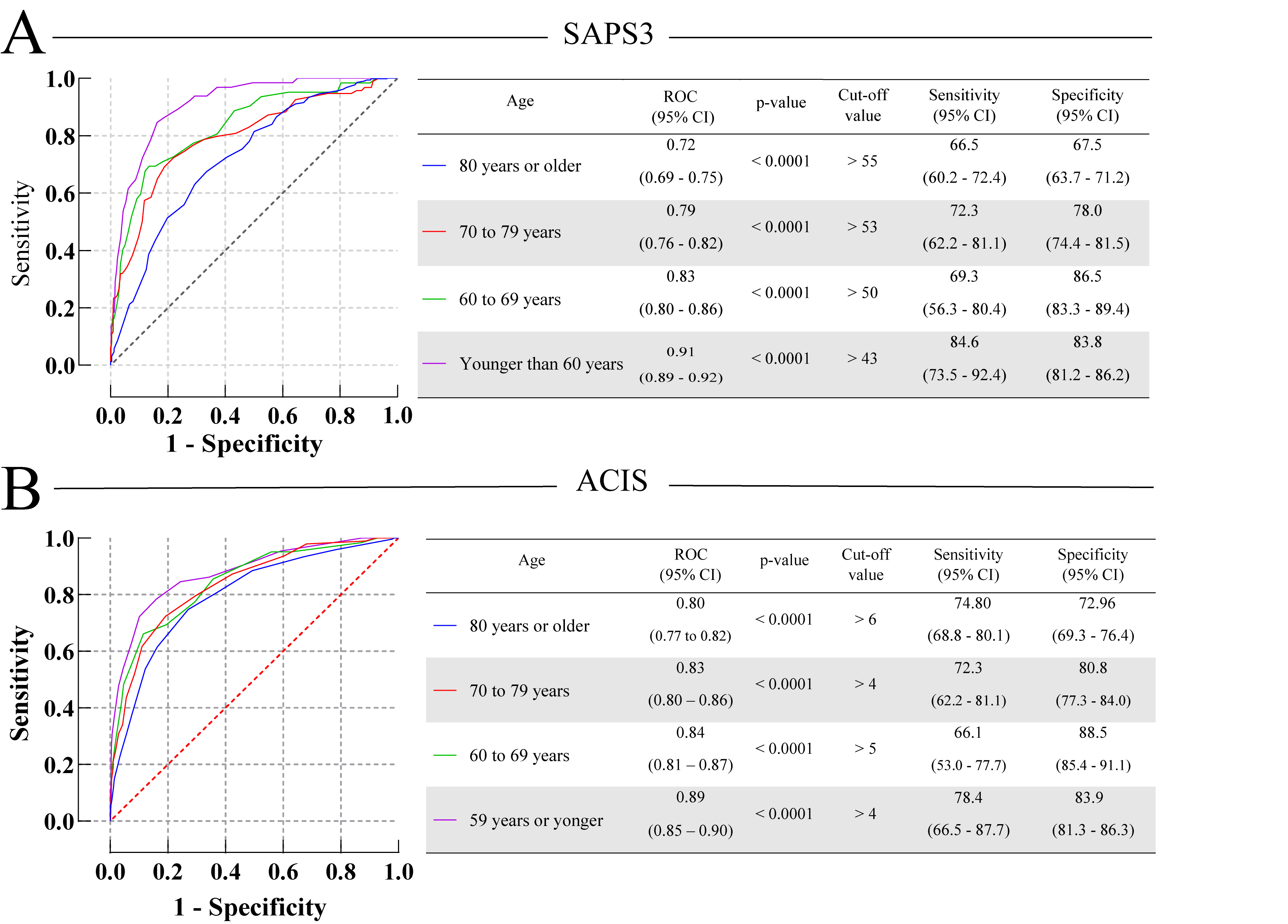

Supplement: S1 Fig — Comparison between SAPS3 (A) and ACIS (B) receiver operating characteristic (ROC) curves stratified by age was performed to test accuracy of the indicated scores in prediction of ICU mortality. Discriminate function of the ACIS was superior to SAPS3 in the 80 years or older subset with comparable performance in the other age interval (TIF) [file pone.0240793.s005.tif]

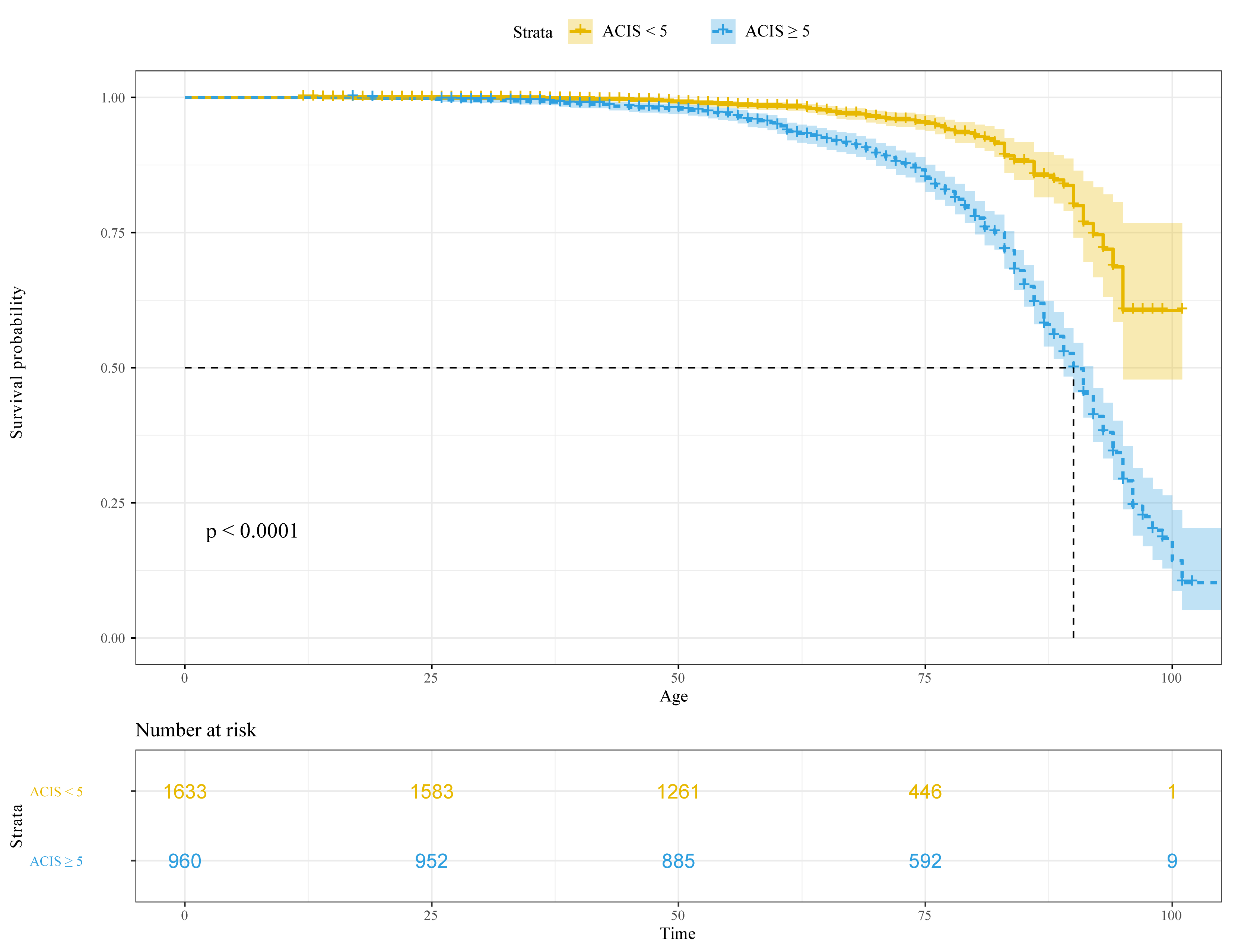

Supplement: S2 Fig — Survival probability is significantly decreased in those over 75 years old with an ACIS score ≥5. (TIF) [file pone.0240793.s006.tif]

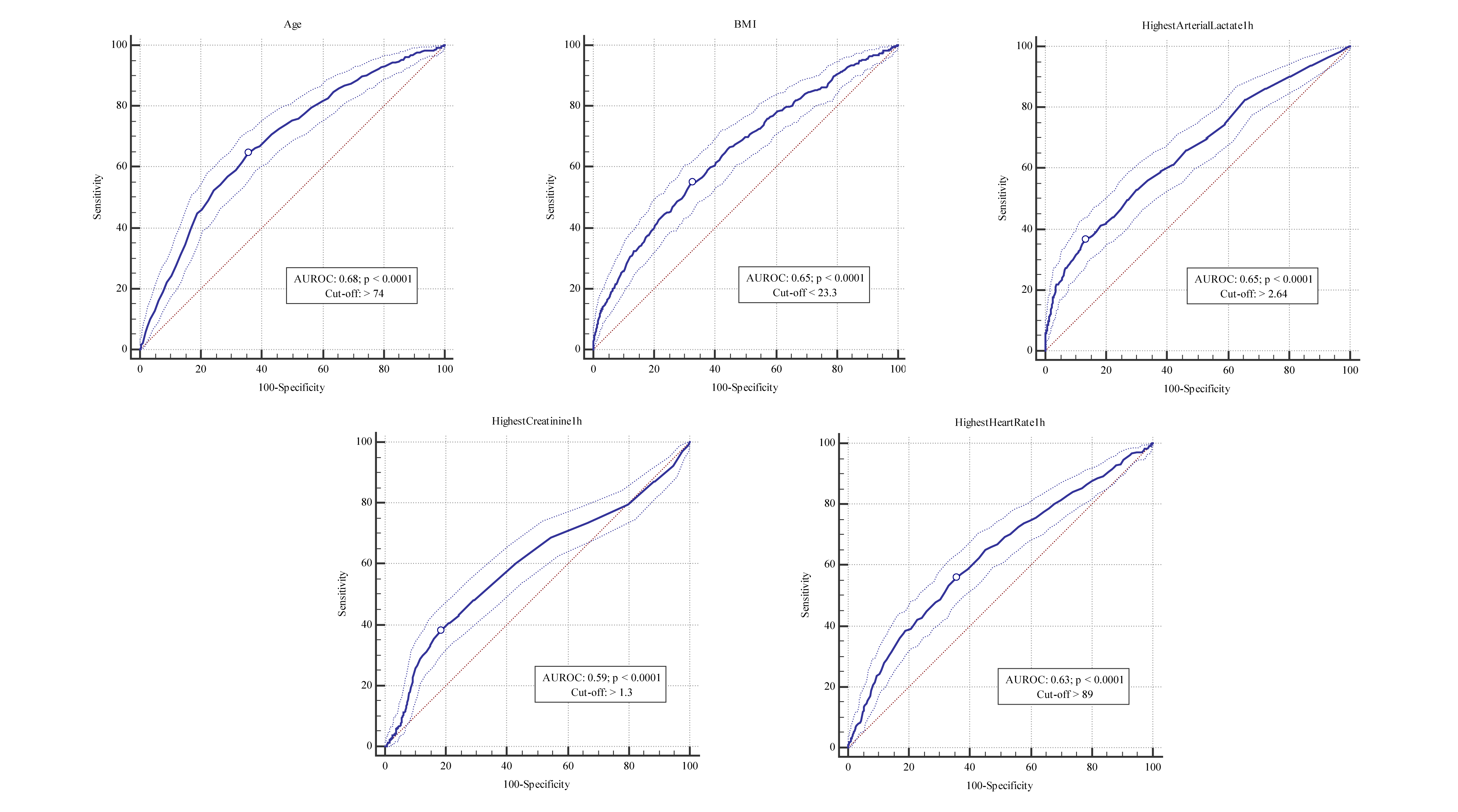

Supplement: S3 Fig — (TIF) [file pone.0240793.s007.tif]
